# Diabetes and all-cause mortality among middle-aged and older adults in China, England, Mexico, rural South Africa, and the USA: a population-based study of longitudinal aging cohorts

**DOI:** 10.1136/bmjdrc-2024-004678

**Published:** 2025-03-18

**Authors:** David Flood, Yuan S Zhang, Emma Nichols, Chihua Li, Paola Zaninotto, Kenneth M Langa, Jinkook Lee, Jennifer Manne-Goehler

**Affiliations:** 1 Department of Medicine, University of Michigan, Ann Arbor, Michigan, USA; 2 Department of Sociomedical Sciences, Mailman School of Public Health, Columbia University, New York, New York, USA; 3 Robert N. Butler Columbia Aging Center, Mailman School of Public Health, Columbia University, New York, New York, USA; 4 Center for Economic and Social Research, University of Southern California, Los Angeles, California, USA; 5 Leonard Davis School of Gerontology, University of Southern California, Los Angeles, California, USA; 6 Institute of Chinese Medical Sciences, University of Macau, Macao, Macao; 7 Survey Research Center, University of Michigan Institute for Social Research, Ann Arbor, Michigan, USA; 8 UCL, London, UK; 9 Department of Internal Medicine, University of Michigan, Ann Arbor, Michigan, USA; 10 Department of Economics, University of Southern California, Los Angeles, California, USA; 11 Division of Infectious Diseases, Massachusetts General Hospital, Boston, Massachusetts, USA

**Keywords:** Internal Medicine, Diabetes Mellitus, Type 2, Geriatrics, Epidemiology

## Abstract

**Objective:**

There is a need for comparable worldwide data on the impact of diabetes on mortality. This study assessed diabetes and all-cause mortality among middle-aged and older adults in five countries.

**Research design and methods:**

We analyzed adults aged 51 years or older followed between 2010 and 2020 from population-based cohorts from China, England, Mexico, rural South Africa, and the USA. The cohorts are part of an international network of longitudinal aging studies with similar sampling designs, eligibility, and assessment methods. Diabetes was defined by self-report or an elevated diabetes blood-based biomarker meeting the clinical criteria for diabetes. All-cause mortality was assessed through linkages or informant interviews. We used Poisson regression models to estimate mortality rate ratios and mortality rate differences, comparing people with diabetes to those without diabetes. Models were adjusted for age, gender, education, smoking status, body mass index, economic status, and, in South Africa, HIV status.

**Results:**

We included 29 397 individuals, of whom 4916 (16.7%) died during the study period. The median follow-up time ranged from 4.6 years in South Africa to 8.3 years in China. The adjusted all-cause mortality rate ratios for people with diabetes versus those without diabetes ranged from 1.53 (95% CI: 1.39 to 1.68) in the USA to 2.02 (95% CI: 1.34 to 3.06) in Mexico. The adjusted mortality rate differences (per 1000 person-years) for people with diabetes vers those without diabetes ranged from 11.9 (95% CI: 4.8 to 18.9) in England to 24.6 (95% CI: 12.2 to 37.0) in South Africa.

**Conclusions:**

Diabetes was associated with increased all-cause mortality in population-based cohorts in China, England, Mexico, rural South Africa, and the USA. Limitations included differences in diabetes biomarkers and selection criteria across cohorts. The results highlight the urgent need to implement clinical and public health interventions worldwide to reduce excess diabetes mortality.

**WHAT IS ALREADY KNOWN ON THIS TOPIC**

- While diabetes has long been associated with increased mortality in high-income countries, contemporary and cross-national estimates of this association have been limited by several factors. We therefore aimed to answer the question of how diabetes impacts all-cause mortality among middle-aged and older adults (aged 51 years or greater) in China, England, Mexico, rural South Africa, and the USA?

**WHAT THIS STUDY ADDS**

- Middle-aged and older adults with diabetes had higher all-cause mortality than people without diabetes in all countries. Relative mortality differences ranged from mortality rate ratios of 1.53 in the USA to 2.02 in Mexico. Absolute mortality differences ranged from mortality rate differences (per 1000 person-years) of 11.9 in England to 24.6 in South Africa.

**HOW THIS STUDY MIGHT AFFECT RESEARCH, PRACTICE OR POLICY**

- There is an urgent need to implement clinical and public health interventions to improve diabetes outcomes globally.

## Introduction

 More than half a billion people worldwide are living with diabetes.^1 2^ By 2050, this number will increase to 1.2 billion people.^1^ Given the epidemiology of diabetes, it is crucial to understand how it impacts long-term health outcomes such as mortality in economically and geographically diverse populations worldwide. All-cause mortality among people with diabetes at the population level is a key metric in the WHO global diabetes monitoring framework.^3^ The WHO recommends monitoring diabetes mortality because it is inherently significant to patients and policymakers, modifiable through evidence-based interventions, and amenable to standardized assessment methods.^3^

While diabetes has long been associated with increased mortality in high-income countries,^4^,^6^ contemporary and cross-national estimates of this association have been limited by several factors. First, there is a paucity of data on diabetes and mortality from low-income and middle-income countries where most people with diabetes live, and this is especially true for middle-aged or older adults who are often understudied in these settings.^7^ Second, temporal declines in all-cause mortality in high-income countries have been observed in recent decades, so updated data are needed.^8^ Third, population data on diabetes and mortality are often not comparable across settings due to differences in sample selection, case definitions, and mortality ascertainment.^7^ These limitations pose challenges for accurately assessing the global burden of diabetes and monitoring diabetes policy responses.

To address these gaps, this study aimed to evaluate the association between diabetes and all-cause mortality among middle-aged and older adults with diabetes using recent data from comparable population-based aging cohorts in five economically and geographically diverse countries.

## Research design and methods

### Study design and sample

We conducted a longitudinal analysis of population-based aging cohorts in five countries: China (China Health and Retirement Longitudinal Study (CHARLS)),^9^ England (English Longitudinal Study of Ageing (ELSA)),^10^ Mexico (Mexican Health and Aging Study (MHAS)),^11^ rural South Africa (Health and Aging in Africa: A Longitudinal Study of an INDEPTH Community in South Africa (HAALSI)),^12^ and the USA (Health and Retirement Study (HRS)).^13^ These cohorts are part of the HRS International Family of Studies, a network of longitudinal aging studies with similar sampling designs, eligibility, and assessment methods.^14^ The cohort inclusion criteria for this analysis were (1) availability of baseline and follow-up data from 2010 to 2020 and (2) collection of a blood-based diabetes biomarker at the baseline wave during this period. We chose 2010–2020 as our period of interest to maximize comparability between cohorts. The cohorts from China, England, and the USA were nationally representative of each country’s middle-aged and older population. The cohort from Mexico was representative from four states (a rural state, an urban state, a state with high migration, and a state with high presumed diabetes prevalence). The cohort from South Africa was representative of rural communities in Southern Africa. See online supplemental appendix 1 for details on the years of data collection and censoring by cohort.

Due to minor differences in the lower end of age eligibility between cohorts, we excluded individuals younger than 51 at baseline to ensure comparability. We also excluded respondents without follow-up information, with no available blood-based diabetes biomarker, or with missing data on prior diabetes diagnosis, gender, education, economic status, smoking status, body mass index (BMI), or survey weights. In the South Africa cohort, we excluded individuals with missing HIV status. Online supplemental appendix 2 shows participant flow diagrams for each cohort including numbers lost to follow-up.

### Definition of diabetes

We defined diabetes as either (1) a history of self-reported diagnosis by a physician or healthcare worker or (2) an elevated blood-based biomarker meeting clinical criteria for diabetes.^15 16^ We used hemoglobin A1c (HbA1c) ≥6.5% (48 mmol/mol) as the biomarker threshold in all countries except China and South Africa, where we used fasting blood glucose ≥126 mg/dL (7.0 mmol/L) or random blood glucose ≥200 mg/dL (11.1 mmol/L). In China, plasma glucose was assessed using an enzymatic colorimetric test (92% of individuals were fasting). In the South African cohort, capillary glucose was assessed using a point-of-care analyzer (24% of individuals were fasting). In England, HbA1c was assessed using venous blood samples. In Mexico, HbA1c was assessed using a point-of-care analyzer certified by the National Glycohemoglobin Standardization Programme.^17^ In the USA, HbA1c was assessed using dried blood spots converted to whole blood equivalent values.^18^ Relevant question text and biomarker details are provided in online supplemental appendices 3 and 4.

### Mortality ascertainment

All-cause mortality was captured in England by linking to the National Health Service Central Register (latest available data from April 2018). In other cohorts, all-cause mortality was captured during interviews with respondents’ spouses or other informants. In all cohorts, the month and year of death were available. If the date of death was unknown, it was estimated as the midpoint between waves in which an individual was known to be alive and had died. We measured survival time in years from the baseline interview as defined in this study to death, loss to follow-up, or the end of the follow-up period (May 2018 in England and December 2019 in the other countries), whichever came first.

### Statistical analysis

Analyses were conducted within each cohort and accounted for survey weights and sampling design when available. We first calculated the overall and age-stratified diabetes prevalence at baseline. In calculating overall prevalence, we age-standardized to the WHO standard population. We then used Poisson regression with an offset for log-transformed person-years and robust standard errors to estimate differences in mortality rate ratios between individuals with diabetes (diagnosed or undiagnosed) and those without diabetes. Poisson models give similar results to Cox models when there are shorter follow-up intervals and have the advantage of directly estimating event rates.^19 20^ Both relative (mortality rate ratios) and absolute (mortality rate and mortality rate differences) measures are reported. Mortality rates and mortality rate differences are presented as the number of deaths per 1000 person-years.

We used prior evidence to develop a directed acyclic graph (DAG) showing our conceptual model of the relationship between diabetes and mortality (online supplemental appendix 5).^21^ While the DAG informed the selection of covariates, some confounders, such as genetic ancestry and physical activity, were unobserved and could not be included in our models. We adjusted for baseline covariates, including age (51–59 years, 60–69 years, and ≥70 years), gender (women vs men), education (less than upper secondary, upper secondary and vocational, and tertiary), smoking status (current vs not current smoker), BMI categories (underweight: BMI <18.5 kg/m^2^; normal weight: 18.5–24.9 kg/m^2^; overweight: 25.0–29.9 kg/m^2^; obese: ≥30.0 kg/m^2^), and economic status (tertiles). Economic status was defined as the annual income of an individual and their coresiding spouse or dependent children in high-income countries (England and the USA), and the annual household per-capita consumption in upper-middle-income countries (China, Mexico, and South Africa). Per-capita consumption is the preferred measure of living standard derived from surveys in the developing countries.^22^ In the South African cohort, we also adjusted for HIV status, given the high prevalence (23%) and known mortality association in this population.^23^

Models were fitted in the overall sample, by age category, and by gender. We also fit a model that separated individuals with diabetes into diagnosed and undiagnosed groups, comparing each to those without diabetes. This analysis aimed to evaluate differences in mortality between individuals with diagnosed diabetes and those with undiagnosed diabetes. Analyses were performed using Stata V.18.0.

### Sensitivity analyses

We conducted three sensitivity analyses. First, we evaluated the sensitivity of our findings to the Poisson assumption of a constant hazard of mortality by fitting alternative survival models (Cox proportional hazard models and Gompertz parametric survival models).^24^ In these models, we used age as the timescale to allow for left truncation, given the current study included participants 51 years and older.^25^ Second, we estimated the association between diabetes and mortality using a slightly more restrictive epidemiological diabetes definition favored by the WHO to reduce misclassification of either (1) the self-reported use of a glucose-lowering medication or (2) an elevated biomarker meeting clinical criteria for diabetes.^26^ Finally, we performed an analysis without the adjustment for BMI categories given the potentially bidirectional relationship between diabetes and BMI.

## Results

### Survey and respondent characteristics

Table 1 presents survey and respondent characteristics for the five cohorts. The final sample included 6251 individuals in China, 4819 in England, 1717 in Mexico, 3411 in South Africa, and 13 199 in the USA. Of the 29 397 total individuals, 4916 (16.7%) died during the study period. The median follow-up time ranged from 4.6 (IQR: 4.4–4.8) years in South Africa to 8.3 (IQR: 8.2–8.4) years in China. There were 191 782 total person-years of follow-up in the cohorts (China: 48 122 person-years; England: 24 536 person-years; Mexico: 11 192 person-years; South Africa: 14 722 person-years; and USA: 93 210 person-years).

**Table 1:** Survey and respondent characteristics

	China (CHARLS)	England (ELSA)	Mexico (MHAS)	South Africa (HAALSI)	USA (HRS)
Survey characteristics					
Years of data collection (baseline to endline)	2011–2019	2012–2018	2012–2019	2014–2019	2010–2019
Sample size, n	6251	4819	1717	3411	13 199
Deaths, n	968	400	206	510	2832
Follow-up time (years), median (IQR)	8.3 (8.2–8.4)	5.5 (5.2–5.7)	7.1 (7.1–7.2)	4.6 (4.4–4.8)	7.5 (6.2–9.0)
Respondent characteristics					
Age (years), median (IQR)	61 (56–68)	64 (57–72)	63 (56–70)	65 (57–73)	63 (57–72)
Women, % (95% CI)	50.2 (47.8–52.6)	50.9 (49.4–52.3)	55.2 (50.8–59.6)	53.7 (52.0–55.4)	54.1 (53.3–54.9)
Education, % (95% CI)					
Less than upper secondary	89.9 (88.2–91.4)	30.8 (29.3–32.5)	88.8 (85.9–91.2)	93.3 (92.4–94.1)	13.9 (12.7–15.4)
Upper secondary and vocational	8.2 (7.3–9.2)	50.7 (49.0–52.4)	3.4 (2.1–5.6)	4.3 (3.7–5.0)	58.7 (56.9–60.4)
Tertiary	1.9 (0.9–3.9)	18.4 (17.1–19.9)	7.7 (5.9–10.1)	2.4 (1.9–2.9)	27.4 (25.5–29.4)
Current smoker, % (95% CI)	31.0 (28.6–33.6)	13.7 (12.5–15.0)	16.9 (12.9–21.8)	8.4 (7.5–9.3)	14.9 (13.8–16.0)
BMI, % (95% CI)					
<18.5 (underweight)	7.5 (6.7–8.3)	0.9 (0.6–1.3)	0.9 (0.4–1.8)	5.6 (4.9–6.5)	1.0 (0.8–1.3)
18.5–24.9 (normal)	61.0 (58.6–63.4)	26.4 (25.0–28.0)	26.0 (21.8–30.7)	36.5 (34.9–38.1)	20.8 (20.0–21.7)
25.0–29.9 (overweight)	27.0 (24.6–29.5)	41.8 (40.2–43.5)	38.0 (34.0–42.2)	28.3 (26.9–29.9)	34.8 (33.8–35.8)
≥30 (obese)	4.6 (3.9–5.3)	30.8 (29.3–32.4)	35.1 (31.0–39.5)	29.6 (28.0–31.1)	43.3 (42.3–44.4)
Diabetes (diagnosed and undiagnosed), % (95% CI)*	15.7 (14.3–17.2)	11.7 (10.6–13.0)	37.4 (33.4–41.5)	12.1 (11.1–13.3)	21.8 (20.8–22.8)
Diagnosed among all with diabetes, % (95% CI)*	46.4 (42.1–50.7)	76.1 (69.3–81.7)	53.6 (47.2–59.9)	57.8 (52.5–62.9)	86.1 (84.1–87.9)

* Values are age-standardized to the WHO standard population among adults aged 50 years and older.

CHARLSChina Health and Retirement Longitudinal StudyELSAEnglish Longitudinal Study of AgeingHAALSIHealth and Aging in Africa: A Longitudinal Study of an INDEPTH Community in South AfricaHRSHealth and Retirement StudyMHASMexican Health and Aging Study

There was considerable cross-country variation in some respondent characteristics, as illustrated in table 1. For example, while nine-tenths of individuals in China (89.9% (95% CI: 88.2% to 91.4%)), Mexico (88.8% (95% CI: 85.9% to 91.2%)), and South Africa (93.3% (95% CI: 92.4% to 94.1%)) had less than an upper secondary education, most individuals in England (69.2% (95% CI: 67.5% to 70.8%)) and the USA (86.1% (95% CI: 84.7% to 87.4%)) had an upper secondary education or greater. Current smoking ranged from 8.4% (95% CI: 7.5% to 9.3%) in South Africa to 31.0% (95% CI: 28.6% to 33.6%) in China. The prevalence of individuals who were obese ranged from 4.5% (95% CI: 3.9% to 5.3%) in China to 43.3% (95% CI: 42.3% to 44.4%) in the USA.

### Diabetes prevalence

**Figure 1:**
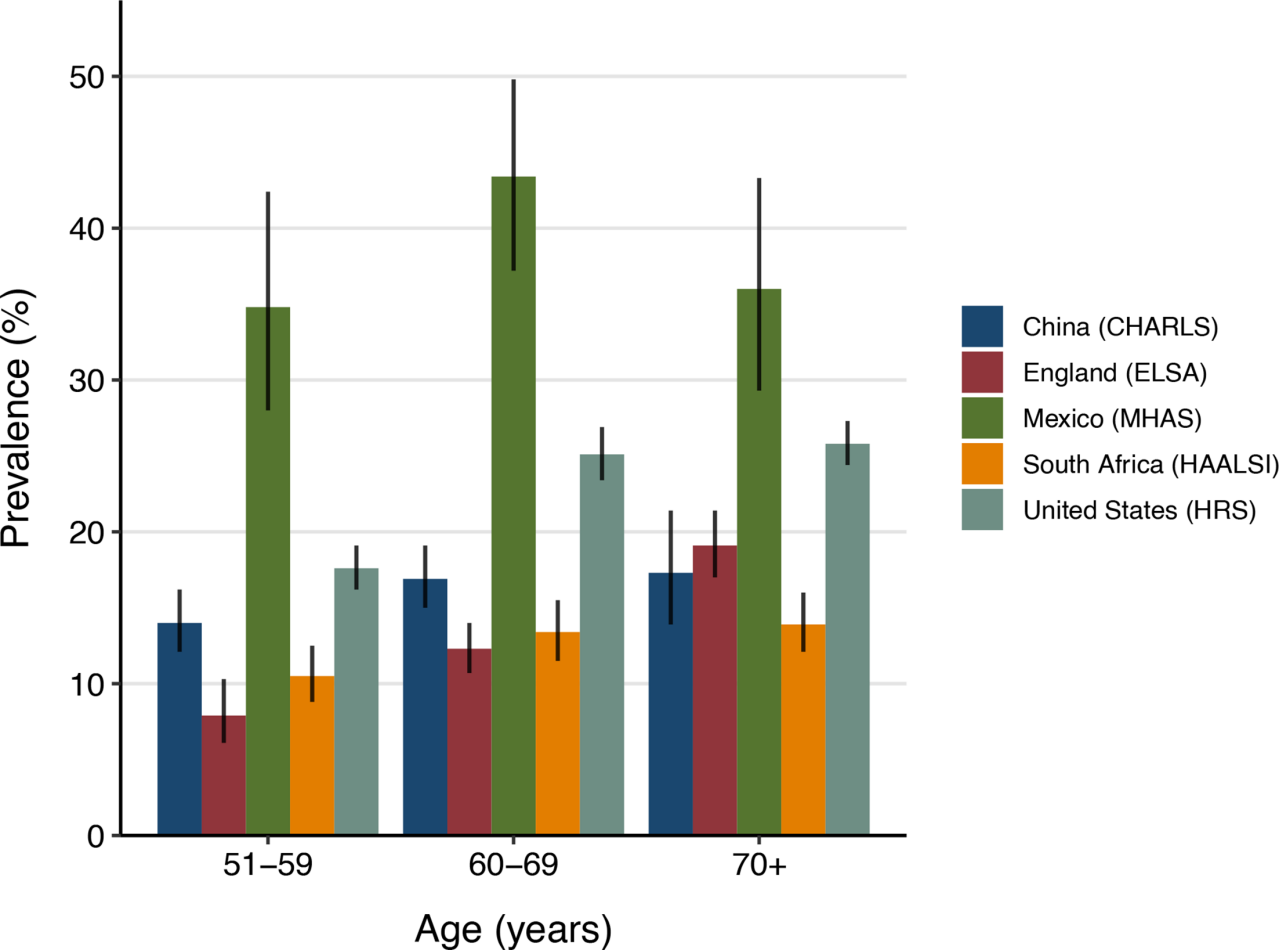
Age-specific prevalence of diabetes by cohort. Diabetes was defined among individuals self-reporting a previous diabetes diagnosis or those with an elevated biomarker (hemoglobin A1c ≥6.5% (48 mmol/mol), fasting plasma glucose ≥126 mg/dL (7.0 mmol/L), or random capillary glucose ≥200 mg/dL (11.1 mmol/L). The vertical error bars represent 95% CIs. CHARLS, China Health and Retirement Longitudinal Study; ELSA, English Longitudinal Study of Ageing; HAALSI, Health and Aging in Africa: A Longitudinal Study of an INDEPTH Community in South Africa; HRS, Health and Retirement Study; MHAS, Mexican Health and Aging Study.

The age-standardized prevalence of diabetes was highest in Mexico (37.4% (95% CI: 33.4% to 41.5%), followed by the USA (21.8% (95% CI: 20.8% to 22.8%)), China (15.7% (95% CI: 14.3% to 17.2%)), South Africa (12.1% (95% CI: 11.1% to 13.3%)), and England (11.7% (95% CI: 10.6% to 13.0%)). Figure 1 shows the age-specific prevalence of diabetes by cohort at baseline. Among individuals with diabetes, the age-standardized proportion of those with diabetes who reported a prior diabetes diagnosis ranged from 46.4% (95% CI: 42.1% to 50.7%) in China to 86.1% (95% CI: 84.1% to 87.9%) in the USA (table 1).

### Mortality rates

**Figure 2:**
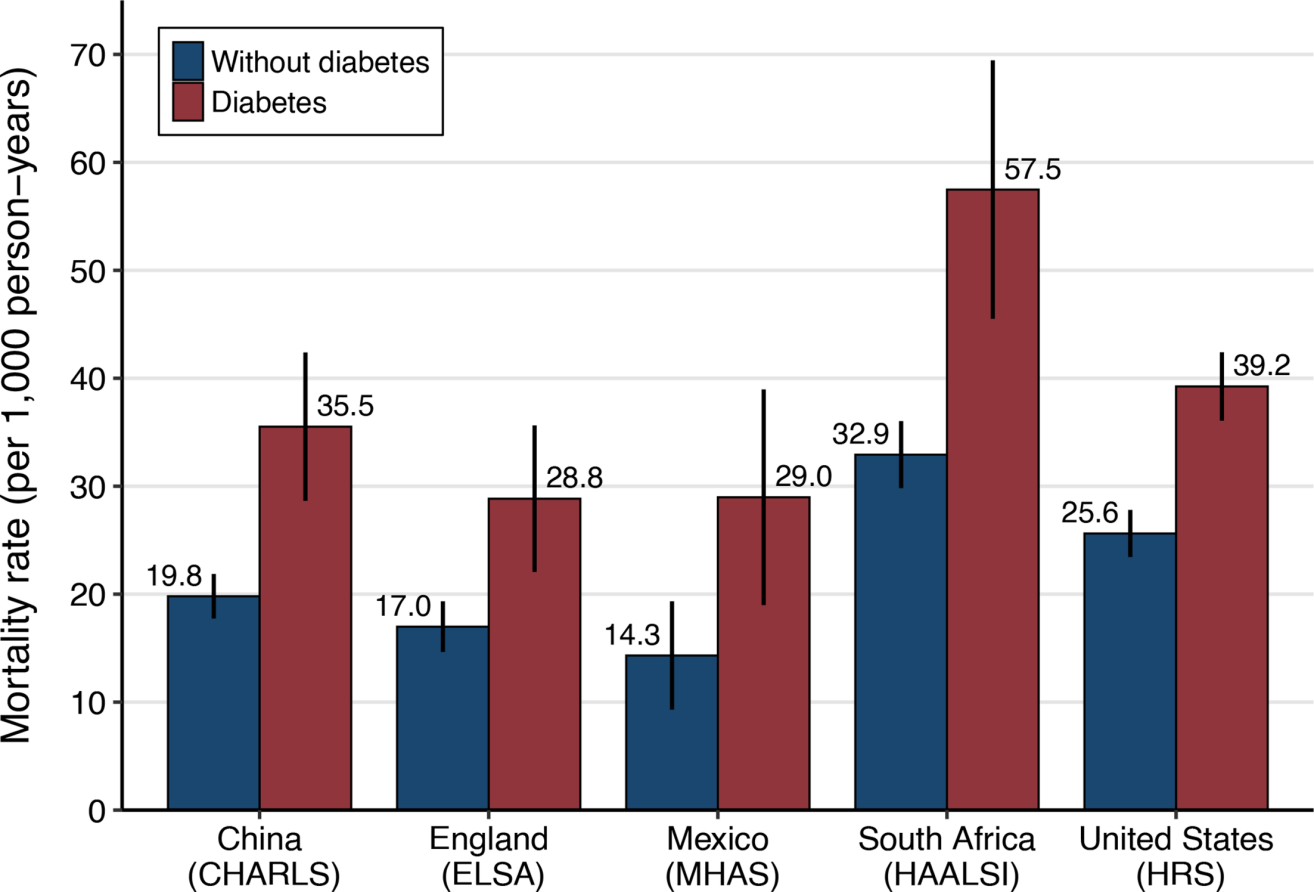
Adjusted all-cause mortality rates by cohort. Mortality rates are presented as the number of deaths per 1000 person-years. The vertical error bars represent 95% CIs. Estimates were derived using Poisson regression models with an offset for log-transformed person-years and robust standard errors and adjusted for age, gender, education, smoking status, body mass index, and economic status. Models in South Africa also adjusted for HIV status. CHARLS, China Health and Retirement Longitudinal Study. ELSA, English Longitudinal Study of Ageing; HAALSI, Health and Aging in Africa: A Longitudinal Study of an INDEPTH Community in South Africa. HRS, Health and Retirement Study; MHAS, Mexican Health and Aging Study.

Adjusted all-cause mortality rates (per 1000 person-years) are presented in figure 2 and online supplemental appendix 6. In each cohort, mortality rates were higher among people with diabetes than those without diabetes. Across the cohorts, mortality rates among people with diabetes were highest in South Africa (57.5 (95% CI: 45.5 to 69.5)), followed by the USA (39.2 (95% CI: 36.1 to 42.4)), China (95% CI: 35.5 (95% CI: 28.6 to 42.4)), England (28.8 (95% CI: 22.1 to 35.6)), and Mexico (29.0 (95% CI: 19.0 to 39.0)).

### Mortality rate ratios and mortality rate differences

**Figure 3:**
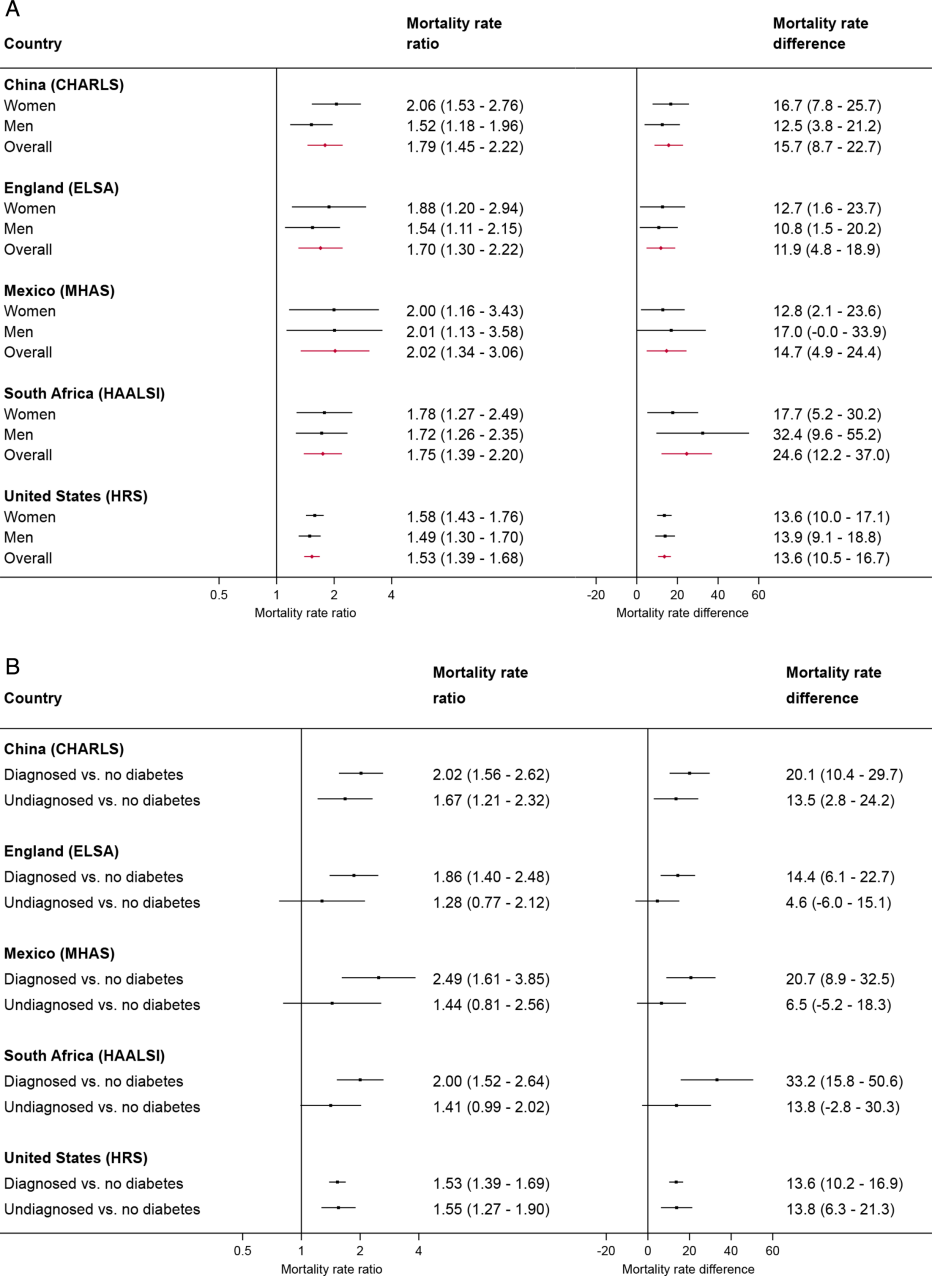
Adjusted all-cause mortality rate ratios and mortality rate differences. (A) Overall and by gender. (B) By diagnosed versus undiagnosed. Mortality rate differences are presented as the number of deaths per 1000 person-years. The horizontal error bars represent 95% CIs. Estimates were derived using Poisson regression models with an offset for log-transformed person-years and robust standard errors and adjusted for age, gender, education, smoking status, body mass index, and economic status. Models in South Africa also adjusted for HIV status. CHARLS, China Health and Retirement Longitudinal Study; ELSA, English Longitudinal Study of Ageing; HAALSI, Health and Aging in Africa: A Longitudinal Study of an INDEPTH Community in South Africa; HRS, Health and Retirement Study; MHAS, Mexican Health and Aging Study.

The adjusted overall all-cause mortality rate ratios for people with diabetes versus those without diabetes ranged from 1.53 (95% CI: 1.39 to 1.68) in the USA to 2.02 (95% CI: 1.34 to 3.06) in Mexico (figure 3A). The adjusted mortality rate differences (per 1000 person-years) for people with diabetes versus those without diabetes ranged from 11.9 (95% CI: 4.8 to 18.9) in England to 24.6 (95% CI: 12.2 to 37.0) in South Africa. No significant differences were observed in adjusted mortality rate ratios or adjusted mortality rate differences by sex in cohorts. Mortality rate ratios appeared to decrease in older age groups in the cohorts from England and the USA (online supplemental appendix 7).

Figure 3B shows results when the diabetes classification was separated by diagnosed or undiagnosed status, compared with no diabetes. In general, there appeared to be a tendency among people diagnosed with diabetes to have higher mortality than people with undiagnosed diabetes. However, these differences were statistically significant only in Mexico, where people with diagnosed diabetes compared with undiagnosed diabetes had an adjusted mortality rate ratio of 1.95 (95% CI: 1.11 to 3.43), corresponding to an adjusted mortality rate difference of 22.6 (95% CI: −7.3 to 52.4) deaths per 1000 person-years (online supplemental appendix 8).

### Sensitivity analyses

The results of the first sensitivity analysis using Cox and Gompertz models (online supplemental appendix 9) were very similar to the main results using Poisson regression models. In the second sensitivity analysis, using the slightly more restrictive epidemiological diabetes definition of either the use of a glucose-lowering medication (instead of self-reported diagnosis) or an elevated biomarker, we observed a slightly higher point estimate for the adjusted mortality rate ratios in the China cohort (1.86 vs 1.79) and slightly lower adjusted mortality rate ratios in the Mexico cohort (1.84 vs 2.02; online supplemental appendix 10). The third sensitivity analysis removing adjustment for BMI had the effect of slightly attenuating the mortality rate ratios and mortality rate differences compared with the main analysis (online supplemental appendix 11).

## Conclusions

In this study of middle-aged and older adults followed between 2010 and 2020 from population-based cohorts in five economically and geographically diverse countries (three of which were nationally representative), we found that people with diabetes consistently had higher all-cause mortality than people without diabetes. Relative mortality differences were similar across cohorts, ranging from mortality rate ratios of 1.53 (95% CI: 1.39 to 1.68) in the USA to 2.02 (95% CI: 1.34 to 3.06) in Mexico. Absolute mortality differences had more variation across cohorts, ranging from mortality rate differences (per 1000 person-years) of 11.9 (95% CI: 4.8 to 18.9) in England to 24.6 (95% CI: 12.2 to 37.0) in South Africa. These findings using recent and comparable data highlight the immense burden of diabetes around the world, particularly in low-income and middle-income countries (represented in our study by China, South Africa, and Mexico), where the absolute mortality impact of diabetes appears greatest. These are also settings where diabetes care is thought to be least robust.^3 27 28^

Many prior studies assessing the association between diabetes and all-cause mortality have been conducted in high-income countries and among younger age groups.^3 7 8^ Large-scale meta-analyses in the last two decades have reported relative mortality differences among people with diabetes, as compared with those without diabetes, that are generally similar to findings in our study.^4 6 29 30^ However, these meta-analyses primarily included non-representative cohorts from high-income countries, limiting population inferences globally. A multicountry analysis from 1995 to 2016 in 16 countries provides updated evidence of a reduction in all-cause mortality among people with diagnosed diabetes, but data were only available from high-income countries.^8^ The Prospective Urban Rural Epidemiology study reported greater absolute mortality among people with diabetes in middle-income and low-income countries, as compared with people with diabetes in high-income countries.^5^ While studies on diabetes-related mortality previously have been performed in each of the countries included in our analysis, including at times using the same underlying cohorts,^31^,^37^ our study uniquely assesses diabetes-related mortality in multiple countries using similar methods across the entire continuum of middle-aged and older adults. Individuals in this age range are sometimes excluded from population-based studies worldwide. However, they have the highest diabetes prevalence and require comprehensive clinical management to prevent diabetes complications.

An important secondary finding in our study was the tendency of higher mortality among people with diagnosed diabetes compared with undiagnosed diabetes. This finding was most marked in Mexico. Many—though not all—prior high-quality population-based studies have reported similar findings.^531^,^33 38^ We hypothesize that the greater mortality among people with previously diagnosed compared with undiagnosed diabetes likely reflects a selection effect related to diabetes severity and/or diabetes duration. Patients with diabetes with the highest disease severity or progression are most likely to experience symptoms, to seek a diagnosis in the healthcare system, and, despite obtaining a diagnosis, to die. This selection effect may be most salient in countries at lower income levels, where the proportion of adults with diabetes who are diagnosed is as low as 20%, compared with 80% or greater in some high-income countries such as the USA.^3^

What are the policy implications emerging from this work? We speculate that the higher absolute mortality rates in South Africa and Mexico are a result of people with diabetes in these countries experiencing challenges accessing quality diabetes care^34 39^ and being impacted by the broader social determinants of health and diabetes.^40^ There is an urgent need to scale up evidence-based interventions to manage diabetes, particularly in low-income and middle-income countries where societies are aging, absolute diabetes mortality is highest, and the population with diabetes is rapidly growing.^2^ Evidence from Sweden shows that people with diabetes who are appropriately managed and achieve risk factor control have little or no excess mortality compared with those without diabetes.^41^ Yet only 10% of people with diabetes in low-income and middle-income countries receive comprehensive diabetes management aligned with guidelines.^27^ In the coming decades, diabetes will cause a staggering degree of premature mortality unless health systems are strengthened to improve diabetes care.^1^ The WHO Global Diabetes Compact is a crucial international effort to stimulate improvements in equitable, affordable, and quality care for people with diabetes.^1 3^ A key pillar of these efforts is the inclusion of stakeholders from the public and private sectors, as well as individuals with lived experiences of diabetes.

Our study has several limitations. First, our analysis did not include people aged 50 years or younger. The younger population with diabetes tends to have a greater hazard of diabetes mortality than the older population without diabetes.^31^,^33^ Our results should not be generalized to the entire population or young population. Still, they can be generalized to the population aged 51 years or older, which represents approximately two-thirds of people with diabetes worldwide.^2^ Second, our use of Poisson models in the main analysis assumes that an individual’s hazard of dying remains constant throughout the observation period, which ranged from a median of 4.6–8.3 years across the five cohorts. We chose this approach because Poisson models allow us to estimate and compare absolute mortality rates directly, expressed as events per person-time, while adjusting for covariates. Furthermore, sensitivity analyses that relax the constant mortality assumption by using Cox and Gompertz models with age as the time scale yielded results consistent with our primary analysis. Third, differences in the blood-based diabetes biomarkers collected in each cohort (eg, glucose vs HbA1c) may contribute to slightly different phenotypes of individuals classified as having undiagnosed diabetes.^42 43^ This limitation could decrease the comparability of estimates across cohorts. As an example of this dynamic, studies in Asian Indians suggest that HbA1c-based diabetes diagnoses may identify individuals with milder glucose intolerance, potentially reflecting less severe disease and lower associated mortality.^44^ Fourth, our study lacks data on cause-specific mortality, preventing us from distinguishing between microvascular and macrovascular patterns of death among individuals with diabetes. Fifth, the Mexican and South African cohorts were not nationally representative, though they were representative of four states in Mexico and a rural community in South Africa like many others in Southern Africa, respectively. Sixth, available cohort data do not allow us to distinguish between type 1 versus type 2 diabetes. Given the age profile of the cohorts, it can be assumed that the vast majority of individuals have type 2 diabetes.^1^ Finally, while this analysis used data from a geographically and economically diverse set of countries, the included cohorts may not fully represent population with diabetes worldwide. In particular, none of the cohorts were drawn from low-income or lower-middle-income countries. Estimating diabetes mortality in these settings is an important area of future research.

In summary, we observed that diabetes was consistently associated with increased all-cause mortality across five diverse settings, and absolute diabetes mortality was particularly high in low-income and middle-income countries, where systems of care for diabetes are known to be weaker. The findings reinforce the need to implement clinical and public health interventions to improve diabetes outcomes in countries worldwide.

## supplementary material

10.1136/bmjdrc-2024-004678 online supplemental file 1

## Data Availability

Data may be obtained from a third party and are not publicly available.
